# Monitoring and modulation of respiratory drive in patients with acute hypoxemic respiratory failure in spontaneous breathing

**DOI:** 10.1007/s11739-024-03715-3

**Published:** 2024-08-29

**Authors:** Anna Mocellin, Federico Guidotti, Simone Rizzato, Matteo Tacconi, Giulia Bruzzi, Jacopo Messina, Daniele Puggioni, Athina Patsoura, Riccardo Fantini, Luca Tabbì, Ivana Castaniere, Alessandro Marchioni, Enrico Clini, Roberto Tonelli

**Affiliations:** 1 Respiratory Diseases Unit, Department of Medical and Surgical Sciences, University Hospital of Modena, University of Modena Reggio Emilia, Modena, Italy; 2 Internal Medicine Unit, University of Rome, Roma 1, Rome, Italy

**Keywords:** Respiratory drive, Sedation, Acute hypoxemic respiratory failure, Non-invasive respiratory support, Monitoring

## Abstract

Non-invasive respiratory support, namely, non-invasive ventilation, continuous positive airway pressure, and high-flow nasal cannula, has been increasingly used worldwide to treat acute hypoxemic respiratory failure, giving the benefits of keeping spontaneous breathing preserved. In this scenario, monitoring and controlling respiratory drive could be helpful to avoid patient self-inflicted lung injury and promptly identify those patients that require an upgrade to invasive mechanical ventilation. In this review, we first describe the physiological components affecting respiratory drive to outline the risks associated with its hyperactivation. Further, we analyze and compare the leading strategies implemented for respiratory drive monitoring and discuss the sedative drugs and the non-pharmacological approaches used to modulate respiratory drive during non-invasive respiratory support. Refining the available techniques and rethinking our therapeutic and monitoring targets can help critical care physicians develop a personalized and minimally invasive approach.

**Graphical abstract:**

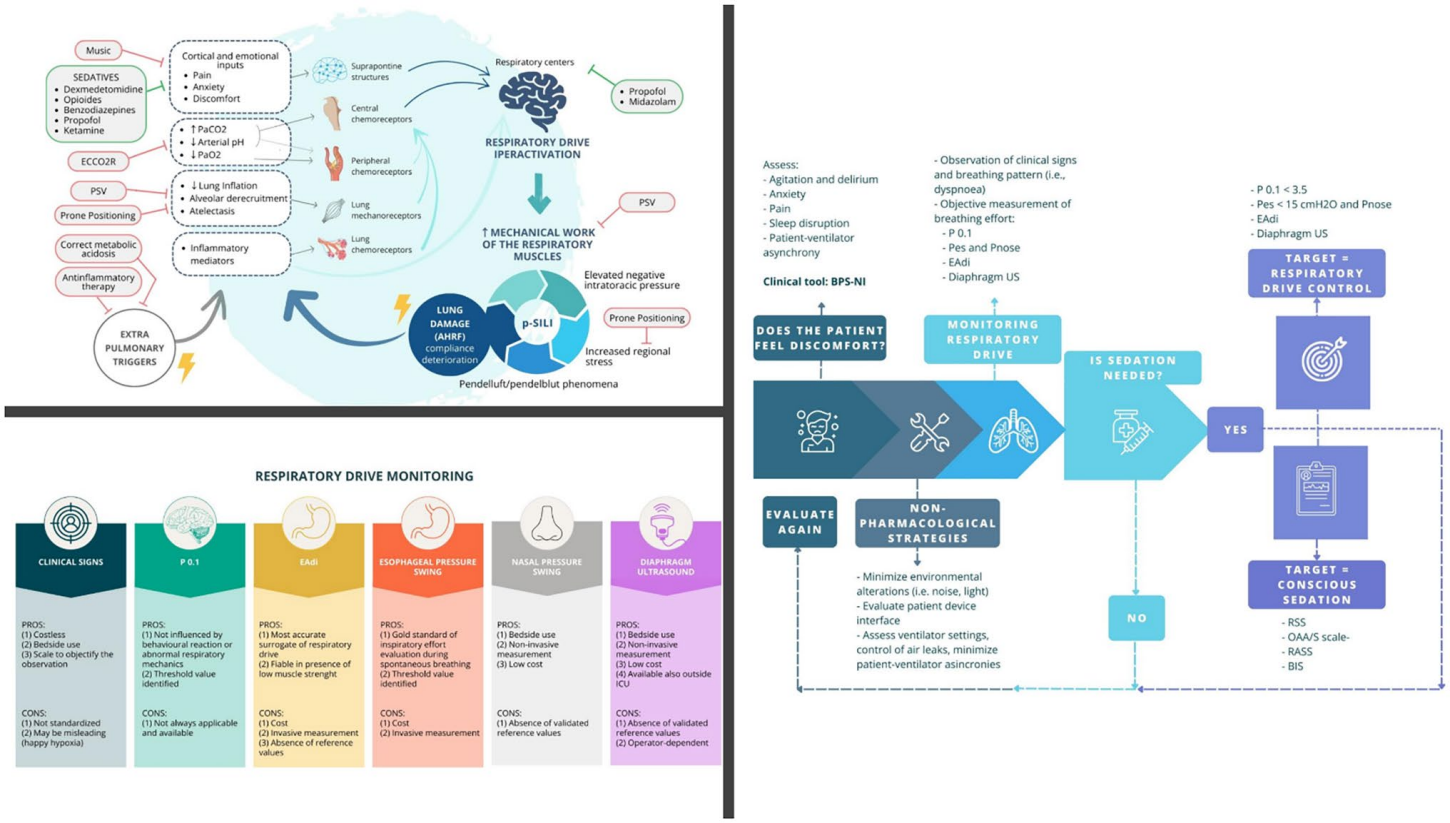

## Introduction

Acute hypoxemic respiratory failure (AHRF) is a life-threatening condition defined by the onset of severe hypoxemia that demands prompt and appropriate management [1]. In recent years, non-invasive respiratory supports (NRS), such as non-invasive ventilation (NIV), continuous positive airway pressure (CPAP), and high-flow nasal cannula (HFNC), are increasingly used as a first step in AHRF treatment, despite protective invasive mechanical ventilation (IMV) remains a cornerstone of the management of patients with severe hypoxemia [2]. The use of NRS has several benefits: it allows the maintenance of spontaneous breathing, thus preserving respiratory muscle function (e.g., it avoids diaphragm dysfunction and atrophy), sparing airways physiology and integrity (e.g., clearance of secretions and cough), and avoiding MV-related complications, such as ventilator acquired pneumonia [3]. Besides, positive end-expiratory pressure (PEEP) improves hemodynamics, ameliorating cardiac pre-loading and cardiac output [4]. On the other hand, spontaneously breathing patients should be accurately monitored to promptly detect NRS failure without delaying the initiation of MV when deemed necessary [5]. In recent years, a new concept has emerged regarding the possible harmful effects of an abnormal activation of respiratory drive in spontaneous breathing AHRF patients. Self-inflicted lung injury (P-SILI) defines a condition of supraphysiological airway pressure and tidal volume (Vt) to which the lung is subjected with the risk of lung damage due to strenuous spontaneous breathing effort [6]. Even though multiple clinical observations and experimental data suggest the existence of SILI, there is currently no certain evidence on the relevance of this physiological phenomenon. However, mitigating excessive respiratory effort in spontaneously breathing patients is becoming the key in the management of many AHRF patients requiring NRS. This review explores the strategies used to monitor and modulate respiratory drive during spontaneous breathing in patients with AHRF. Most evidence derives from studies and models having acute respiratory distress syndrome (ARDS) as a paradigm. However, AHRF and ARDS appear to belong to the same disease spectrum portrayed by lung injury, hypoxemia, altered respiratory mechanics and alveolar dead space fraction, and increased respiratory drive [3, 7, 8].

## Physiology of respiratory drive

Respiratory drive is commonly defined as the intensity of the output of the respiratory centers, determining the mechanical work of the respiratory muscles, i.e., breathing effort [9]. Recently, Jonkman et al. proposed a more accurate and comprehensive definition of respiratory drive: the time integral of the neuronal network of the respiratory centers, derived from estimates of breathing effort [10]. This concept includes the evaluation of amplitude, frequency, or both of neural activity [10]. The respiratory drive determines breathing effort only if neuromuscular transmission and respiratory function are preserved. The neuronal centers located in the medulla and pons receive tonic inputs from different sources to regulate the three phases of the respiratory cycle: inspiration, post-inspiration, and expiration [11]. The complex web of interconnection interacting and modifying respiratory activity is still partly unknown. Cortical and emotional inputs, such as pain, anxiety and discomfort, may affect both the brain curve (independently from the patient’s metabolic demands) and the respiratory drive through behavioral responses and a direct reflex on medullary respiratory centers [12, 13]. Chemical feedback is determined by central and peripheral receptors. The first ones, located in the medulla oblongata, are highly susceptible to pH and PaCO_2_ of the cerebrospinal fluid and directly modulate the frequency and intensity of the respiratory center’s output [14]. The second ones, located in the carotid bodies and also influenced by PaO_2_, stimulate breathing pattern by enhancing the threshold sensitivity of the central chemoreceptors [15]. Severe hypoxemia can stimulate the peripheral chemoreceptors that increase the neural respiratory drive by improving the ventilatory response to CO_2_. This mechanism can be further enhanced by concomitant hypercapnia (for example, caused by increased dead space) and altered pH that stimulates central and peripheral chemoreceptors [16]. It is important to note that peripheral chemoreceptors well tolerate mild hypoxemia, being significantly activated by a severe drop in blood PaO_2_. Thus, the most relevant blood gas parameter in the regulation of respiratory drive is PaCO_2_ [7].

Mechanical inputs, determined by lung stretch receptors and activated by lung inflation, inhibit central chemoreceptors, terminating inspiration [9]. When lung damage occurs with associated atelectasis and alveolar de-recruitment, lung mechanoreceptors’ inhibitory reflex can be reduced, enhancing the output of the respiratory centers [10].

Inflammatory mediators that activate vagal C-fibers increase respiratory drive. The inflammation occurring during a systemic disease (e.g., sepsis or ARDS) improves the sensitivity of peripheral chemoreceptors to hypoxemia, with stimulation of lung chemoreceptors (C-fibers) and respiratory centers directly by cytokine production [17].

All the mechanisms described above can be illustrated by two curves:

*-the brain curve* that expresses the minute ventilation requested by the neural respiratory drive for a given PaCO_2;_
*-the ventilation curve* that describes the actual minute ventilation of the subject for a given PaCO_2_ [7].

If the respiratory flow–generation pathway (from the neural cells to the respiratory muscles) is intact, the brain curve is identical to the ventilation curve. To clarify, “demand equals supply”: the levels of PaCO_2_ requested from the brain show a linear correlation with the Vt that the respiratory system can guarantee to deliver a linear correlation [11].

## Factors influencing respiratory drive during AHRF

During AHRF, impaired neuromuscular function and abnormal respiratory system mechanics generate a dissociation between the brain and the ventilation curves [11]. The resulting PaCO_2_ at a given level of respiratory drive is higher than that expected by the brain as the respiratory generation pathway is impaired at different levels [7]. The ventilation curve is influenced by the respiratory drive, the respiratory rate, the integrity of the inspiratory flow–generation pathway, the ventilator setting, and the patient–ventilator interaction [11].

Spontaneous breathing limits diaphragm atrophy and dysfunction, permits earlier mobilization, and improves hemodynamics [6, 18, 19]. On the other hand, the high uncontrolled ventilatory drive promotes elevated breathing effort with detrimental effects on lungs and diaphragm [20]. In AHRF patients, high respiratory drive leads to great inspiratory effort, local alveoli over-distention, and negative pressure pulmonary edema [21]. The cyclic recruitment of dependent lung zones and the inhomogeneous transmission of stress and strain worsen P-SILI [10]. In ARDS animal model, inspiratory effort generates an inhomogeneous distribution of transpulmonary pressure variation across the lung, with a greater pressure change in the dependent regions (posterior) than in non-dependent ones (anterior). The result of this uneven distribution of forces during spontaneous breathing is the so-called “pendelluft phenomenon,” which corresponds to an intrapulmonary shift of gas from non-dependent to dependent lung regions without a change in V at the very onset of the inspiratory effort. The consequence is a selective overinflation of dependent regions and simultaneous deflation in the non-dependent lung area, reproducing a mechanism that promotes lung damage through a zonal volutrauma and cyclic opening/closing of the dependent regions (i.e., atelectrauma). Furthermore, significant inspiratory effort, during assisted ventilation, can cause a drop in alveolar pressure below the PEEP, resulting in aggravation of pulmonary edema due to an increase of transvascular hydrostatic pressure. This deleterious mechanism is amplified in case of low ventilatory assistance and high airways resistance. Even though excessive inspiratory effort could theoretically result in worsening of pre-existing alveolar damage, Yoshida et al., in an elegant experimental study, demostred that a self-inflicted lung damage occurs only in severe lung injury; however, in mild lung injury spontaneous breathing may be accompained by an improvement in alveolar damage and respiratory mechaincs. Transposed to the clinical setting, the concept of P-SILI suggests the need for monitoring inspiratory effort especially in spontaneusly breathing patients suffering from severe AHRF undergoing NRS. Furthermore, in addition to the well-known harmful effects on the lung parenchyma, MV can also injure the diaphragm, resulting in muscle dysfunction which is defined as “myotrauma.” Despite it is known that the muscle disuse, as occurs in controlled MV, triggers proteolytic pathways that result in diaphragm atrophy and contractile dysfunction. More recent evidence suggests that myotrauma may also be the result of excessive loading of the muscle. Clinical and experimental studies show that contraction against an excessive load leads to acute diaphragm inflammation and weakness; however, relieve inspiratory loading significantly attenuates muscle fiber injury in an experimental sepsis model. The increase in diaphragm thickness, measured by ultrasound (US), in patients undergoing assisted IMV, is associated with impaired diaphragm function and prolonged MV, introducing the concept of underassistance myotrauma. All these observations suggests that in AHRF patients with hyperactivation of the respiratory drive, a self-inflicted diaphragm injury, due to excessive loading of the muscle, may also be present. A complex approach that combines the achievement of lung and diaphragm protective strategies, the adjustment of ventilation parameters, and the titration of sedation is required to prevent the development of such a harmful condition [18]. Low respiratory drive should also be avoided because of its potential adverse consequences, which include progressive atrophy of the diaphragm due to weak inspiratory effort, patient–ventilator asynchronies, and sleep fragmentation [10, 11].

## Strategies for respiratory drive monitoring

During AHRF, the respiratory flow–generation pathway could be affected at different levels according to the disease’s etiology. As the output of the respiratory centers cannot be directly measured, it is essential to identify the best monitoring surrogate of the respiratory drive. To this purpose, various indices of motor and neural output can be determined [22, 23] as shown in Table 1.

**Table 1:** Respiratory drive monitoring tools: Pros and Cons

	Clinical signs	P.01	EAdi	Esophageal manometry	Nasal manometry	Diaphragm ultrasound
Pros	1) Bedside use 2) Non-invasive 3) Scales to objectify the observation	1) Not influenced by behavioral reaction or abnormal respiratory mechanics 2) Threshold value identified	1) Most accurate surrogate of respiratory drive 2) Reliable in presence of low muscle strength	1) Gold standard for inspiratory effort assessment 2) Threshold value identified	1) Bedside use 2) Non-invasive 3) Low cost 4) Available outside ICU	1) Bedside use 2) Non-invasive 4) Available outside ICU
Cons	1) Not standardized 2) May be misleading (e.g., “happy hypoxia”)	1) Not always applicable and available	1) High cost 2) Invasive 3) Absence of validated reference values	1) High cost 2) Invasive	1) Absence of validated reference values	1) Absence of validated reference values 2) Operator dependent

*P 0.1* airway occlusion pressure, *EAdi* diaphragm electrical activity

Early identification of markers and signs of excessive activation of respiratory drive is necessary to assume appropriate ventilatory and pharmacologic strategies and promptly detect NRS failure [18].

Assessing respiratory drive starts with bedside clinical evaluations. A common symptom during AHRF is dyspnea, directly linked to high drive activation. Dyspnea is the result of multiple sensory feedback from chemoreceptors and mechanoreceptors and depends on the integrity of sensory information (further modified by emotions like anxiety and pain) and the motor answer [24, 25]. Dyspnea is often considered the clinical result of the discrepancy between the desired ventilation (brain curve) and the actual ventilation achievable (ventilation curve) [7]. It could be measured with scales and scores (e.g., Borg or Visual Analogue Scale) [26]. However, as patients may be non-responsive or uncooperative, it can be helpful to objective signs of dyspnea and increased inspiratory effort [27]. A valuable indicator to observe is the tracheal tug, characterized by the downward motion of the trachea with each inspiratory effort. While the degree of tug may differ among patients, its presence is consistently meaningful as the respiratory muscles induce tugging when the diaphragm pulls the entire mediastinum downward during each inspiratory effort [28]. Another clinical sign is the assessment of the sternomastoid muscle. Phasic contraction of the sternomastoid is frequently observed in patients with acute respiratory failure, and it is associated with a forced expiratory volume in the first second less than half that observed in patients without such contraction [29, 30]. Lastly, the inspection of the suprasternal fossa can also be helpful. As swings in pleural pressure (P_Pl_) become more negative, the suprasternal fossa is visibly excavated with each inspiration. This excavation is directly proportional to swings in esophageal pressure (P_es_) [31]. Even if shortness of breath is frequently associated with severe hypoxemia, some clinical conditions leading to AHRF may lack dyspnea and clinical signs of inspiratory effort [32]. The recent SARS-CoV-2 pandemic has revealed that some patients may not manifest dyspnea even in severely reduced PaO_2_ because of the “happy hypoxemia” phenomenon. In this kind of patients, during the initial phase of the illness, there is no increased airway resistance and dead space ventilation, so the lung’s compliance is substantially preserved, and the breathing effort seems to remain unchanged [33]. As such, instrumental methods for evaluating and estimating respiratory drive may help clinicians in detecting harmful hyperactivation of the respiratory drive.

One of the most accurate methods to assess the inspiratory effort is measuring the airway occlusion pressure (*P*0.1), defined as the negative airway pressure developed in the first 100 ms during the inspiratory phase developed against an airway occlusion [34]. In spontaneously breathing mechanically ventilated patients, a value above 3.5 cm H_2_O indicates a high respiratory drive, thus reflecting a vigorous respiratory muscle contraction [35]. It is not influenced by behavioral reaction (the usual time reaction is superior to 150 ms) nor abnormal respiratory mechanics [34]. However, it is unreliable in severe respiratory muscle weakness, and there is no evidence about its utility during NRS [36].

Assessing the diaphragm electrical activity (EAdi) may provide an accurate estimate of the breathing effort. To perform the measurement, an esophageal catheter with multiple electrodes measures the change in the discharge of motor neurons to the diaphragm over time [37]. Hence, it is the most accurate surrogate of respiratory drive, even in low muscle strength. However, the invasiveness of the measurement, the low availability of intensive care unit (ICU) ventilators able to record it, and the absence of normal reference values [38] significantly reduce its use in clinical practice.

To date, esophageal manometry pressure with *P*_es_ swing (∆*P*_es_) assessment is considered the gold standard for the inspiratory effort evaluation during spontaneous breathing [18]. *P*_es_ represents an accurate surrogate of the *P*_Pl_ that allows the calculation of the inspiratory transpulmonary pressure during static condition in patients undergoing IMV, providing a reliable measure of the lung stress. During spontaneous breathing ∆*P*_es_ coincides with the dynamic transpulmonary pressure, while in assisted MV, the dynamic transpulmonary pressure is affected by pressure support (PS) and PEEP as well as the inspiratory effort measured as ∆*P*_es_. While the dynamic transpulmonary pressure may represent the global stress applied to the lung parenchyma during assisted and un-assisted spontaneous breathing, some clinical observations show that the inspiratory effort (i.e., ∆*P*_es_) is the most important component associated with P-SILI. Currently, there is no evidence that allows us to estabish a harmful threshold of ∆*P*_es_; however, values over 10–12 cm H_2_O might be considered a threshold risk for P-SILI development, and its monitoring over time could be very useful in the early identification of NRS failure [39]. The major limitations of this technique are the invasive monitoring (it requires a nasogastric tube with an esophageal balloon), the high cost, and the need for specific expertise in performing the calibration and the measurements [40].

Nasal pressure swings (∆*P*_nose_) is a physiological variable that reflects the airway pressure (*P*_aw_) swings captured in the upper respiratory tract during tidal breathing. It has been recently demonstrated that ∆*P*_nose_ has a strong correlation with ∆*P*_es_ regardless tha application of HFNC or NIV. In contrast to ∆*P*_es_, ∆*P*_nose_ can be easily measured at the patient’s bedside with a “nasal plug” inserted in the nostril, not influencing inspiratory effort or respiratory rate [41]. Recently, in a real-life cohort of patients with AHRF undergoing HFNC, ∆*P*_nose_ showed high accuracy in predicting early NRS failure [42].

US could be helpful in monitoring inspiratory effort [43]. Vivier et al. found a significant correlation between the thickening fraction (TF) as assessed by US and the diaphragmatic pressure–time product per breath (PTPdi per breath = average inspiratory pressure × time/number of breaths) in 12 patients treated with NIV with three increasing PS levels following extubation [44]. Further, Umbrello et al. found a significant correlation between TF and Esophageal Pressure–Time Product (PTP_es_) and P0.1 [45], in a population of patients who met the criteria for a spontaneous breathing trial with pressure support ventilation (PSV) following major elective surgery. Although US may provide an accurate estimate of breathing effort, it is an operator-dependent technique, making it challenging to reproduce. Further, no reference cutoff has been identified.

## Pharmacological modulation of respiratory drive

The strict monitoring of respiratory drive aims at its modulation, after signs of hyperactivation are detected. As mentioned, P-SILI is supposed to occur during spontaneous breathing secondary to high respiratory drive. Hence, keeping the breathing effort and respiratory rate within the physiological threshold may reduce the risk of further lung damage [46]. For this reason, applying a sedative strategy aimed at controlling respiratory drive could improve NRS success rate and thus reduce the need for IMV. This concept has been introduced by Kassis et al. [47] with the name of “lung-protective sedation,” based on the interaction between patient and ventilator, to target synchrony instead of arousal. In this case, sedation should be evaluated by direct measures of synchrony and effort.

Important to note is that sedation in hypoxemic spontaneously breathing patients is still perceived as an insidious issue and unstandardized practice, leading to very limited use in daily routine (between 25 and 40% of patients) [48].

To date, the use of sedative drugs in AHRF patients under NRS has consistently aimed to improve interface tolerance. As HFNC is per se better tolerated than NIV [49] to our knowledge, there are no studies that have investigated the use of sedative drugs in patients with AHRF treated with HFNC. Conversely, one of the most frequent causes of premature interruption of NIV is mask intolerance due to pain, discomfort, delirium, or claustrophobia [50, 51].

Ideally, sedation in hypoxemic patients should be performed with no/minimal respiratory depression and no/minimal impairment of the upper airways, maintaining the patient easily arousable [52, 53]. In this regard, Yang and colleagues conducted a meta-analysis to assess the clinical efficacy of using sedative and analgesic drugs during NIV. They concluded that the use of sedative drugs in this subset of patients reduces the intubation rate and delirium and shortens the duration of stay in the ICU [54].

From a pharmacological point of view, sedatives may directly dampen the respiratory drive [10]. However, sedative regimens are usually titrated based on scales assessing the neurological status of the patients (i.e., arousal), such as the Richmond Assessment Sedation Scale (RASS), Riker Sedation–Agitation Scale (SAS), and Ramsay Sedation Scale (RSS) [55]. However, available data suggest that arousal level poorly correlates with patient respiratory effort and ventilator synchrony [56, 57]. Moreover, clinical studies based on hypoxemic patients on NRS aimed at assessing the impact of sedation on respiratory effort as the primary outcome are lacking. The most used drugs for sedation during NIV are dexmedetomidine, opioids, benzodiazepines, and propofol. The characteristics of each agent are discussed below and summarized in Table 2.

**Table 2:** Pharmacological synopsis

	Dexmedetomidine	Remifentanil	Propofol	Midazolam	Ketamine
Mechanism of action	Short-acting alpha 2 adrenoceptor agonist	Short-acting opioid with a µ-selectivity	GABAA receptors activation	GABAA receptors activation	NMDA antagonistic action
Doses used during NIV	Bolus: 1 µg/kg (optional) Maintenance: 0.2–0.7 µg/kg/h Step up-down: 0.1 µg/kg/h	Maintenance: 0.025 µg/kg/min Step up-down: 0.01 µg/kg/min every minute	Maintenance: 0.4 µg/mL Step up-down: 0.2 µg/mL	Bolus: 0.05 µg/kg Maintenance: 0.05–0.1 µg/kg/h Step up-down: 0.05 µg/kg/h	No data during NIV
Respiratory drive modulation	Minimal	Moderate	Elevated	Elevated	Minimal
Sedative effect	Moderate	Moderate	Elevated	Elevated	Moderate
Analgesia	Moderate	Elevated	Minimal	Minimal	Elevated
Downsides	Bradycardia Hypotension Hemodynamic depression	Risk of accumulation Nausea and vomiting Hemodynamic depression Chest wall rigidity	Respiratory depression Hemodynamic instability Airway instability and aspiration	Accumulation Paradoxical agitation Amnesia Delirium	Salivary secretions Nausea and vomiting Altered mental status Visual hallucinations

*GABA*_*A*_ γ-aminobutyric acid type A*, NMDA* N-methyl-D-aspartic acid, *NIV* non-invasive ventilation

### Dexmedetomidine

Dexmedetomidine is a short-acting selective α_2_-adrenergic agonist that stimulates receptors located in the locus coeruleus to provide sedation and anxiolysis [58]. Further, it acts on the spinal cord to enhance analgesia without significant respiratory depression. It also causes sympatholysis via central and peripheral mechanisms [59]. In animal models, there is increasing evidence that dexmedetomidine can provide protective effects for the lungs exposed to acute damage through anti-inflammatory, anti-apoptotic, and antioxidant properties [60]. The effects of dexmedetomidine on the respiratory system resulted in minimal changes in respiratory frequency and a slight reduction in minute ventilation, leading to a modest increase in PaCO_2_ [61]. In an observational study conducted on 33 spontaneously breathing patients, sedation with dexmedetomidine did not result in changes in the diaphragmatic TF measured by diaphragmatic US [62]. Compared with any sedation strategy (in particular with remifentanil and propofol) or placebo during NIV, dexmedetomidine has shown a better profile regarding intubation rates, delirium, ICU length of stay, and length of NIV. There were no significant differences in all-cause mortality [59, 63, 64]. The most reported adverse reactions in patients receiving dexmedetomidine are hypotension, hypertension, and bradycardia (occurring in approximately 25, 15, and 13% of patients, respectively), generally resolved with no treatment [65].

### Opioids

Opioids have been historically used for sedation during NIV [66], even though they can cause concentration-dependent hypoventilation and increased irregularity of breathing [67]. In a prospective observational cohort study, 12 adult patients received a continuous sufentanil infusion at 0.2 to 0.3 micro g x kg^−1^ × hr^−1^ during PSV [68]. Sufentanil infusion did not affect respiratory drive measured through P0.1.

Remifentanil, a short-acting opioid with μ-selectivity, is widely used for the sedation of critically ill patients with AHRF [69]. Used as a single sedative agent, it allows to obtain the desired level of awake sedation with little effects on minute volume, respiratory pattern, blood gases, and hemodynamics compared to other opioids [70]. Low doses of remifentanil generate a slight decrease in the patient’s respiratory rate without significant changes in Vt and respiratory drive, as quantified by P0.1 [71]. EAdi was assessed in thirteen intubated patients who were administered increasing doses of remifentanil during PSV [72]. The authors showed that remifentanil did not modify EAdi but only respiratory timing. Remifentanil seems to obtain a more significant reduction of respiratory rate than dexmedetomidine; thus, the effect on minute ventilation is more appreciable [73]. Moreover, remifentanil seems to have a superior analgesic effect compared to dexmedetomidine [74]. Observational studies showed that sedation with remifentanil has resulted feasible and safe during NIV [75, 76]. However, to our knowledge, no randomized controlled trials (RCT) have ever been conducted to assess its use in spontaneously breathing patients with AHRF.

### Propofol

Propofol is a short-acting intravenous anesthetic that positively modulates the inhibitory function of γ-aminobutyricacid (GABA) type A (GABA_A_) receptors and leads to central nervous system depression, resulting in sedation and anesthesia [77]. Clouzeau and colleagues conducted an observational study on ten adult patients sedated with target-controlled infusion (TCI) of propofol during poorly tolerated NIV with good results [78]. The very low concentration used allowed patient cooperation and did not compromise spontaneous respiration, ensuring an effective and safe technique. In one case, excessive respiratory depression was observed. Interesting data about the influence of propofol on respiratory drive arise from a prospective crossover RCT conducted by Vaschetto and colleagues [79]. During PSV, increasing the depth of sedation with propofol determined a progressive significant decrease in neural drive (measured through electrical activity of the diaphragm) and respiratory effort (∫ electrical activity of the diaphragm/min). However, deep propofol sedation increased patient–ventilator asynchronies, while light sedation did not [80].

### Benzodiazepines

Benzodiazepines are molecules that enhance the effect of the neurotransmitter GABA at the GABA_A_ receptor, resulting in sedative, hypnotic, and anxiolytic effects [81]. Benzodiazepines affect respiration in several ways. First, they modulate the muscular tone, leading to an increased risk of upper airway obstruction; further, they flatter the ventilatory response curve to carbon dioxide. Indeed, benzodiazepines dampen the respiratory reaction to hypoxia, while hypercapnia has occurred [82].

The effects of midazolam on respiratory muscles at a dosage of 0.1 mg/kg were studied in nine healthy volunteers. After infusion, the ratio of gastric pressure (*P*_ga_) on *P*_es_ changes (Δ*P*_ga_/ Δ*P*_es_ index) significantly decreased, indicating reduced diaphragmatic activity [83]. Flumazenil can reverse this effect, as confirmed by the measurement of EAdi after its administration in patients sedated with midazolam [84].

In the past, benzodiazepines were one of the most used pharmacological classes for sedation practices of patients on NIV [85]. Its use is currently limited due to its low safety profile and poor handling. Among benzodiazepines, midazolam is one of the most used drugs, showing hypnotic, sedative, and amnestic properties [86]. However, compared to dexmedetomidine, it showed worse outcomes in NIV sedation, such as the duration of mechanical ventilation and the length of the ICU stay [87, 88].

### Ketamine

Ketamine is a non-competitive antagonist of the N-methyl-D-aspartic acid (NMDA) receptor that can induce a state of “dissociative anesthesia.” Ketamine is an excellent analgesic drug, similar to opioids but with a lower incidence of respiratory drive depression [89]. It is also an ideal agent for maintaining homeostasis (cardiovascular stability, maintenance of respiratory reflexes), especially in patients who require ongoing maximal sympathetic activity [90]. Ketamine has a minimal impact on controlling respiratory centers; however, it may be effective in achieving control of respiratory drive through indirect mechanisms [91]. To date, no studies have assessed this sedative’s effectiveness and safety profile during NRS.

## Non-pharmacological control of respiratory drive

**Fig. 1:**
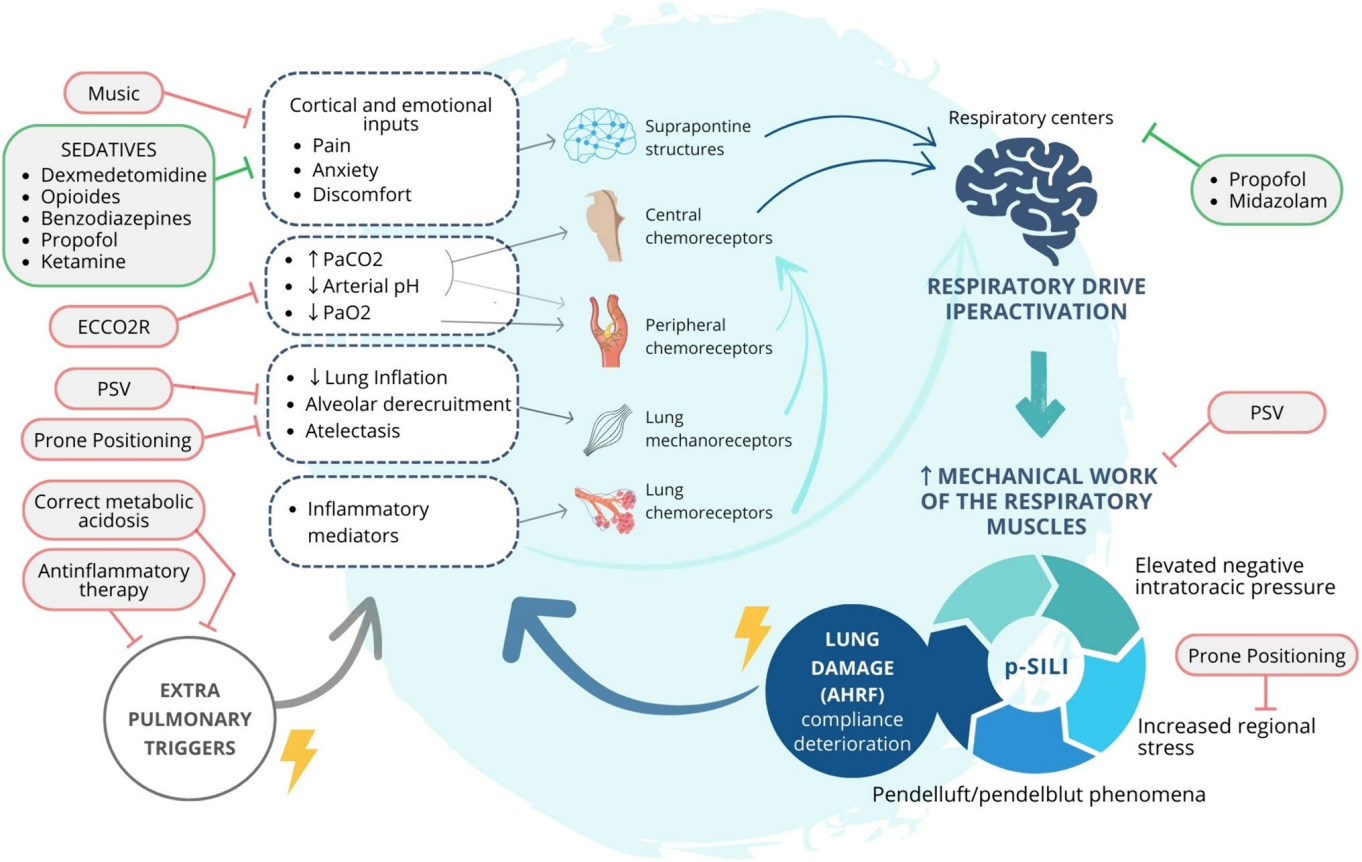
Schematic representation of the causes of hyperactivation of the respiratory drive and the pharmacological and non-pharmacological options to control them. This figure represents the vicious cycle that can be triggered by lung damage leading to AHRF. Cortical, biochemical, mechanical, and inflammatory stimuli result in hyperactivation of the respiratory drive, leading to an increase in the mechanical work of respiratory muscles, thus initiating a vicious cycle that culminates in the formation of P-SILI. Non-pharmacological possibilities are mentioned in the pink boxes, while pharmacological options are listed in the green boxes to control this cycle. *AHRF* acute hypoxemic respiratory failure*; P-SILI* patient self-inflicted lung injury, *PSV* pressure support ventilation, *ECCO*_*2*_*R* extracorporeal carbon dioxide removal

High respiratory drive and its causes should be addressed to adjuvate pharmacological measures to prevent lung and diaphragm injuries. As mentioned before, non-respiratory factors may increase respiratory drive. In this line, pain, discomfort, metabolic acidosis, fever, and other precipitating factors should be promptly identified and corrected [3]. Besides sedation, clinicians might consider non-pharmacological strategies aimed at preserving respiratory drive activation within the physiological threshold. To provide the patient the highest comfort while being assisted with NRS includes the rotation of interfaces, optimizing ventilatory settings to improve ventilator–patient interaction, and the management of anxiety and pain. Non-pharmacological strategies aimed at maximizing the control of respiratory drive are briefly illustrated below and summarized in Fig. 1

### Music

The use of relaxing music might be considered a low-cost, side-effectless option to control anxiety and its consequences on respiratory drive [92]*.* Classical music [92] or relaxing music [93] seem to be the most appropriate choices for this type of hospital setting. The main modalities in which it can be used are music therapy, conducted by certified music therapists, and therapeutic music listening, administered by nurses.

Music positively influences ICU inpatients in the physiological, psychological, and social spheres [92], and it is likely to reduce anxiety and depression and improve sleep quality [94].

A RCT has described a favorable interaction between music rhythm and the breathing pattern of critically ill subjects receiving ventilatory support [93]. Indeed, music seemed able to override metabolic inputs by decreasing anxiety and increasing comfort, thus dampening and decreasing the behavioral drive [7]. Conversely, another RCT focused on respiratory comfort during NIV recently failed to demonstrate a beneficial impact of musical intervention compared to conventional care [95]. Further studies are warranted on the interaction between music and respiratory drive [94]. No study has been conducted on the impact of music in modulating respiratory effort in ICU patients with AHRF and supported through NRS.

### Awake prone position

The prone position (PP) was first proposed for patients with ARDS in the 1970s [96], later developed within a multimodal approach of such pathology [97].

Physiological effects of pronation include changes in inflation, ventilation, and perfusion, permitting decompression of the dorso-caudal dependent zone. The increased functional residual capacity and the homogeneous inflation and perfusion result in reduced lung stress and, thus, in a lowering of the respiratory drive hyperactivation [98, 99]. Awake prone position (APP) improves diaphragmatic function and reduces inspiratory effort [99].

During the recent COVID-19 pandemic, the so-called “awake pronation” in non-invasive mechanically ventilated patients was often performed. This practice is feasible and has been associated with a reduction in intubation rate [100], especially in patients undergoing HFNC [101], and work of breathing during CPAP [98]. High-quality evidence available is RCT derived from studies enrolling only COVID-19 patients in non-intubated patients [100, 102].

Regarding the respiratory drive, Whatheral et al. [102] noted seven trials reporting changes in respiratory rate, but significant heterogeneity in the timing outcome assessment precluded the pooling of data for statistical analysis.

According to available literature, the use of this practice outside the respiratory intensive care unit (R-ICU) or ICU setting should be discouraged [99]. In patients in conventional oxygen therapy (COT) who do not receive NRS-type respiratory support, the practice of pronation remains controversial [99, 100]. Available studies show dyshomogeneity in the duration of pronation, with variability between 1 and 12 h [100, 101]; however, it appears that the impact of the practice is related to the duration of APP [99]. More data are needed on the effect of APP in non-COVID-19 patients with AHRF. Indeed, ESICM 2023 task force was unable to make a recommendation for or against awake for patients with non-COVID19 AHRF [103]. Further research is warranted to explore the effect of APP on mortality, inspiratory effort, and work of breathing in non-COVID-19 patients with AHRF [99].

### Extracorporeal carbon dioxide removal

Extracorporeal carbon dioxide removal (ECCO_2_R) aims to reduce the amount of CO_2_ via an extracorporeal circuit: this will move the metabolic hyperbola downward, thus reducing the current PaCO_2_ and minute ventilation level [104]. The primary endpoint of ECCO_2_R in ARDS is to reduce the injury due to mechanical ventilation. Crotti et al. [105] described an innovative approach using extracorporeal membrane oxygenation in awake spontaneously breathing patients: CO_2_ removal relieved work of breathing and permitted extubation in many patients (bridge to lung transplant or affected by Chronic Obstructive Pulmonary Disease), only a few patients with ARDS were able to perform the spontaneous breathing trial. To date, the burden of ECCO_2_R-related complications is too high to consider this method to reduce the respiratory drive in non-intubated patients with mild ARDS [103].

## Adequate setting and respiratory support

The use of sedative drugs during NRS in spontaneously breathing patients with AHRF should be limited to physician and nurses with experience in management of sedative therapy and its adverse effects, with adequate patient monitoring, in a high-intensity setting such as R-ICU or ICU. This implies the need for adequately trained staff and good resource availability. It is also important to emphasize that, to reduce respiratory drive, these drugs should always be considered adjuncts to a respiratory support system (HFNC or NIV), which still plays a primary role in this context. It is not certain that all approaches proposed for AHRF in the ICU are reproducible in alternative settings, either in terms of patient safety or efficacy (i.e., awake pronation [99]). There is a lack of studies regarding sedation in patients with AHRF undergoing NRS that compare multiple medications and different approaches to respiratory support. High-flow nasal oxygen (HFNO) is the currently suggested first-line intervention [2], but the optimal non-invasive management of AHRF is still debated. New evidence is emerging that is shedding light on the type of patient who would benefit the most from non-invasive ventilatory support to reduce respiratory effort activation [106, 107].

## Needs for research and further perspective

**Fig. 2:**
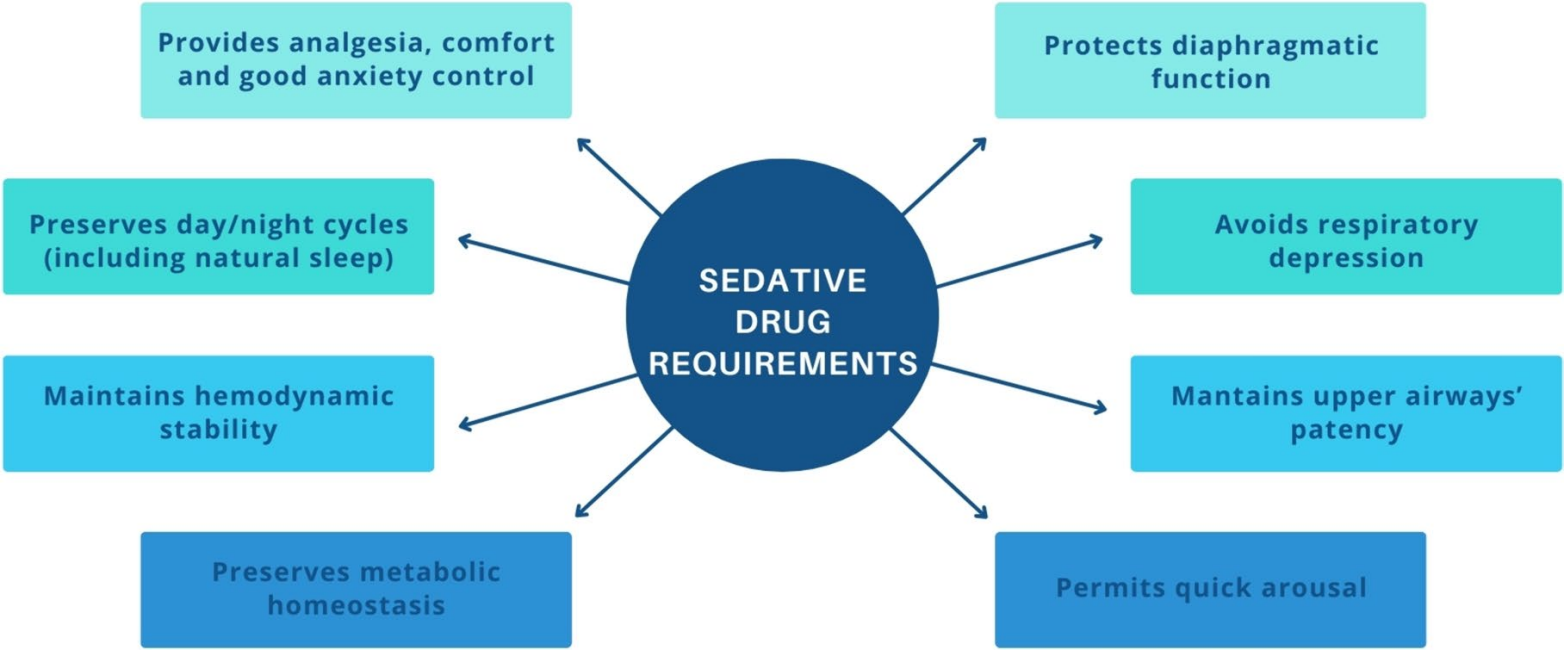
Characteristics of the ideal sedative drug

A notable concern is the need for RCTs and comparative effectiveness studies among the currently available sedative drugs. The ideal sedative drug to be used in spontaneously breathing patients with AHRF should not only dampen but should also preserve ventilatory drive, keep safe effects on airway patency, avoid the onset of delirium, promote natural sleep, have a low impact on hemodynamics, and produce anxiolysis (Fig. 2). Additionally, considerations should extend to the drug’s economic viability, environmental sustainability, and ease of implementation in healthcare settings. Presently, no specific drug fully meets all these criteria.

Including patients receiving HFNO therapy in RCTs seems imperative, as the indication for AHRF is now clearly established and included in the new ARDS definition [2, 8]. High tolerance and ease of use of HFNO could facilitate the shift of analog sedation from a method focused on improving patient tolerance and ventilation synchrony to one aimed at preventing P-SILI onset.

Most importantly, it should be assessed whether reducing respiratory drive in patients exhibiting overactivation can decrease P-SILI and, consequently, prevent technique failure and the need for increased invasiveness.

**Fig. 3:**
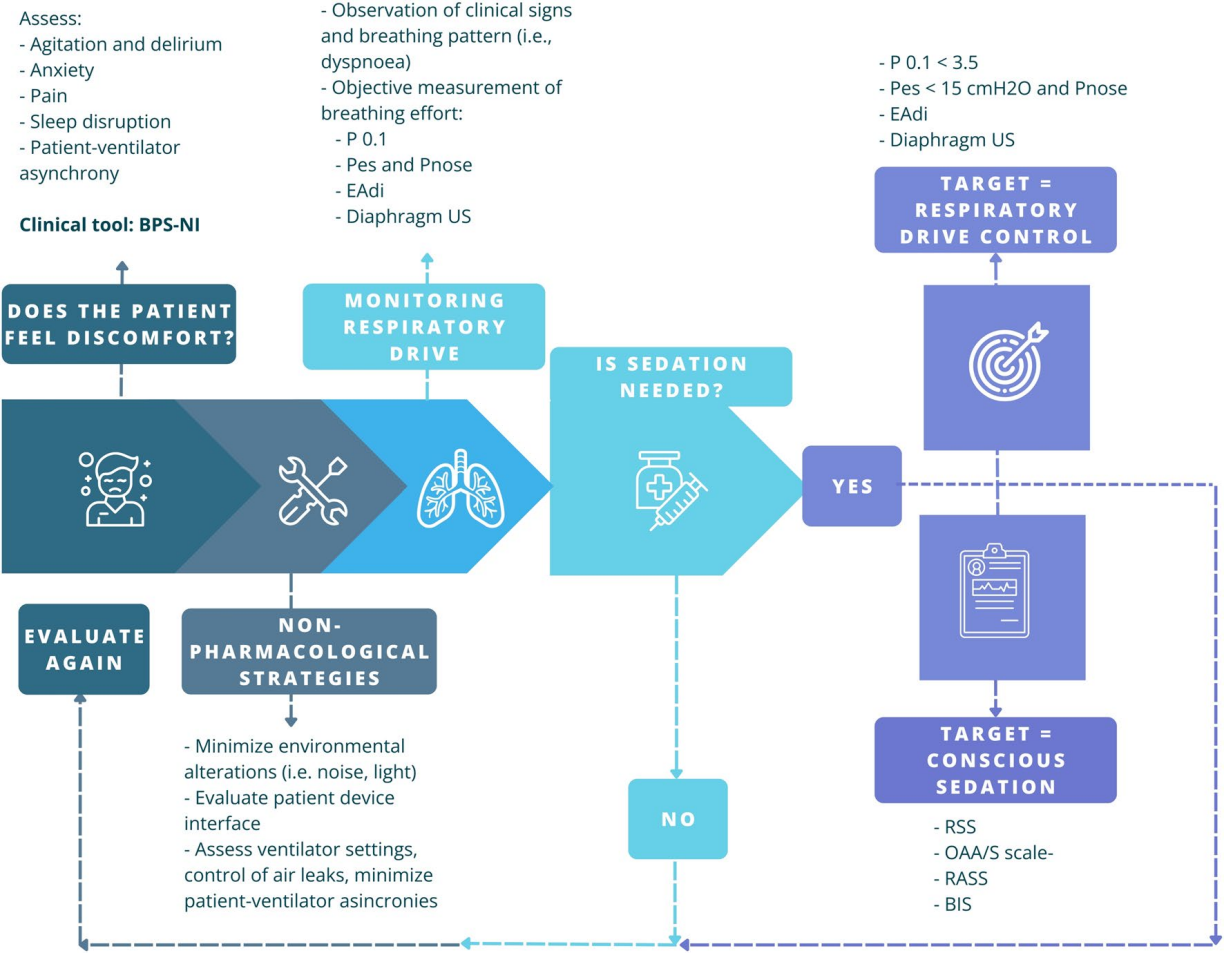
“Lung-protective sedation” model. Preliminary assessment: search for signs and symptoms of discomfort and implement non-pharmacological strategies to reduce them. By integrating the preliminary assessment and measuring respiratory drive, it is possible to decide whether sedation is needed or not. If sedation is initiated, it is necessary to achieve the correct level of sedation and control of the respiratory drive through close monitoring. *P0.1* Airway occlusion pressure, *ΔP*_es_ Esophageal pressure swings, *ΔP*_nose_ Nasal pressure swings, *EAdi* Diaphragm electrical activity, *BPS-NI* Behavioral pain scale non-intubated patients, *US* ultrasound, *RSS* Ramsay sedation scale, *OAA/S* observer’s assessment of alertness/sedation, *RASS* Richmond assessment sedation scale, *BIS* bispectral index

Considering monitoring, sedation, and proper respiratory support choice as not dissociable pillars of the management of spontaneously breathing AHRF patients would allow for identifying and attaining a safe level of inspiratory effort. This could form the basis for a new concept of “protective non-invasive respiratory support” (Fig. 3). This process, in turn, necessitates the concurrent advancement of minimally invasive and cost-effective techniques for monitoring inspiratory effort, enabling the identification of the subset of patients who would benefit from such an approach. Artificial intelligence (AI) will likely play a pivotal role in integrating data, vital parameters, and sedation levels to enhance the monitoring of non-invasive respiratory support.

## Conclusions

The use of NRS has recently surged to manage patients with AHRF [2]. Keeping spontaneous breathing preserved requires clinicians to forecast potential consequences of P-SILI through close monitoring of inspiratory effort and respiratory drive. Clinical patient evaluation focused on respiratory rate and accessory muscle involvement is feasible but lacks objectivity. The ideal tool to quantify the activation of the respiratory drive should balance non-invasiveness, low cost, and reproducibility. In this line, diaphragmatic US and ∆P_nose_ assessment seem promising techniques. Once signs of hyperactivation are detected, a pharmacological approach to dampen respiratory drive is welcomed. In this scenario, dexmedetomidine appears to have the best risk–benefit profile as a sedative drug for pain, discomfort and anxiety control, and delirium prevention. A (i.e., selecting the appropriate NRS mode, APP, and interface rotation in case of NIV). Further evidence is needed to enable a more standardized procedure in the NRS setting. The integrated approach of the methods examined should aim at a protective, non-invasive respiratory support strategy modeled upon the profile of the patient’s inspiratory effort.

## Data Availability

Not applicable.

## Abbreviations

AHRF: Acute hypoxemic respiratory failure
AI: Artificial intelligence
APP: Awake prone position
ARDS: Acute respiratory distress syndrome
BIS: Bispectral index
BPS-NI: Behavioral pain scale non-intubated patients
COT: Conventional oxygen therapy
CPAP: Continuous positive airway pressure
EAdi: Diaphragm electrical activity
ECCO_2_R: Extracorporeal carbon dioxide removal
GABA: γ-Aminobutyric acid
GABA_A_: γ- Aminobutyric acid type A
HFNC: High-flow nasal cannula
HFNO: High-flow nasal oxygen
ICU: Intensive care unit
IMV: Invasive mechanical ventilation
MV: Mechanical ventilation
NIV: Non-invasive ventilation
NMDA: N-methyl-D-aspartic acid
NRS: Non-invasive respiratory support
OAA/S: Observer’s assessment of alertness/sedation
P0.1: Airway occlusion pressure
P_aw_: Airway pressure
PEEP: Positive end-expiratory pressure
P_es_: Esophageal pressure
P_ga_: Gastric pressure
P_Pl_: Pleural pressure
PP: Prone position
PS: Pressure support
P-SILI: Patient self-inflicted lung injury
PSV: Pressure support ventilation
PTPdi: Diaphragmatic pressure–time product
PTPes: Esophageal pressure–time product
RASS: Richmond assessment sedation scale
R-ICU: Respiratory intensive care unit
RCT: Randomized controlled trial
RSS: Ramsay sedation scale
SAS: Riker sedation–agitation scale
TCI: Target-controlled infusion
TF: Thickening fraction
US: Ultrasound
Vt: Tidal volume
∆P_es_: Esophageal pressure swing
∆P_nose_: Nasal pressure swings
